# Aged dissolved organic carbon exported from rivers of the Tibetan Plateau

**DOI:** 10.1371/journal.pone.0178166

**Published:** 2017-05-26

**Authors:** Bin Qu, Mika Sillanpää, Chaoliu Li, Shichang Kang, Aron Stubbins, Fangping Yan, Kelly Sue Aho, Feng Zhou, Peter A. Raymond

**Affiliations:** 1 Laboratory of Green Chemistry, Lappeenranta University of Technology, Mikkeli, Finland; 2 Department of Civil and Environmental Engineering, Florida International University, Miami, Florida, United States of America; 3 Key Laboratory of Tibetan Environment Changes and Land Surface Processes, Institute of Tibetan Plateau Research, Chinese Academy of Sciences, Beijing, China; 4 State Key Laboratory of Cryospheric Sciences, Norwest Institute of Eco-Environment and Resources, Chinese Academy of Sciences, Lanzhou, Gansu, China; 5 CAS Center for Excellence in Tibetan Plateau Earth Sciences, Chinese Academy of Sciences, Beijing, China; 6 Skidaway Institute of Oceanography, Department of Marine Science, University of Georgia, Savannah, Georgia, United States of America; 7 Yale School of Forestry and Environmental Studies, Yale University, New Haven, Connecticut, United States of America; 8 Sino-France Institute of Earth Systems Science, Laboratory for Earth Surface Processes, College of Urban and Environmental Sciences, Peking University, Beijing, China; University of Connecticut, UNITED STATES

## Abstract

The role played by river networks in regional and global carbon cycle is receiving increasing attention. Despite the potential of radiocarbon measurements (^14^C) to elucidate sources and cycling of different riverine carbon pools, there remain large regions such as the climate-sensitive Tibetan Plateau for which no data are available. Here we provide new ^14^C data on dissolved organic carbon (DOC) from three large Asian rivers (the Yellow, Yangtze and Yarlung Tsangpo Rivers) running on the Tibetan Plateau and present the carbon transportation pattern in rivers of the plateau versus other river system in the world. Despite higher discharge rates during the high flow season, the DOC yield of Tibetan Plateau rivers (0.41 gC m^-2^ yr^-1^) was lower than most other rivers due to lower concentrations. Radiocarbon ages of the DOC were older/more depleted (511±294 years before present, yr BP) in the Tibetan rivers than those in Arctic and tropical rivers. A positive correlation between radiocarbon age and permafrost watershed coverage was observed, indicating that ^14^C-deplted/old carbon is exported from permafrost regions of the Tibetan Plateau during periods of high flow. This is in sharp contrast to permafrost regions of the Arctic which export ^14^C-enriched carbon during high discharge periods.

## Introduction

Global river export of dissolved organic carbon (DOC) to the oceans is a key component of the global carbon cycle and is subject to climate forcing [1]. After entering into aquatic environment, a significant fraction of the DOC will be deposited or redeposited temporarily and stored in floodplains or reservoirs or degraded by photochemical and biological degradation processes [1–3]. As subsidies of the terrestrial sources, DOC concentrations in aquatic system are potentially driving increasing by global warming and anthropogenic activities [4, 5].

Recent evidence suggested that there is a significant, and selective degradation of old bioavailable terrestrial organic carbon (1000 to >21000 years before present, yr BP) in the high-latitude polar ecosystems [6]. Similar observations also reported from the rivers and lakes in northern temperate regions and tropics of northern Australia [7, 8], and thus highlighting the biological link between ancient sources of carbon and contemporary aquatic carbon biogeochemistry.

With the global warming, polar ecosystems are experiencing the most dramatic warming in the world [9], and huge amount of ancient carbon stored in the permafrost are releasing into rivers [9–12], much of which will be rapidly degraded to carbon dioxide [3]. Similar to that in Arctic regions, most of the Tibetan Plateau, the ‘Third Pole’ and the highest and largest plateau on earth, is also underlain by permafrost, and the permafrost soils on the Tibetan Plateau is as well potentially vulnerable to climate warming [13]. It was proposed that the permafrost active layer thickness (ALT) on the Tibetan Plateau had increased by 0.15 to 0.50 m from 1996 to 2001 due to climate change [13]. Therefore, with a huge carbon store of 12.3 Pg-C (1 Pg = 10^15^ g) on the Tibetan Plateau [13] and more than ten large rivers (e.g. Yellow River, Yangtze River, Yarlung Tsangpo, etc.) running on this region [14], old soil carbon might be released by the hydrological changing in this climatic-sensitive area.

**Fig 1:**
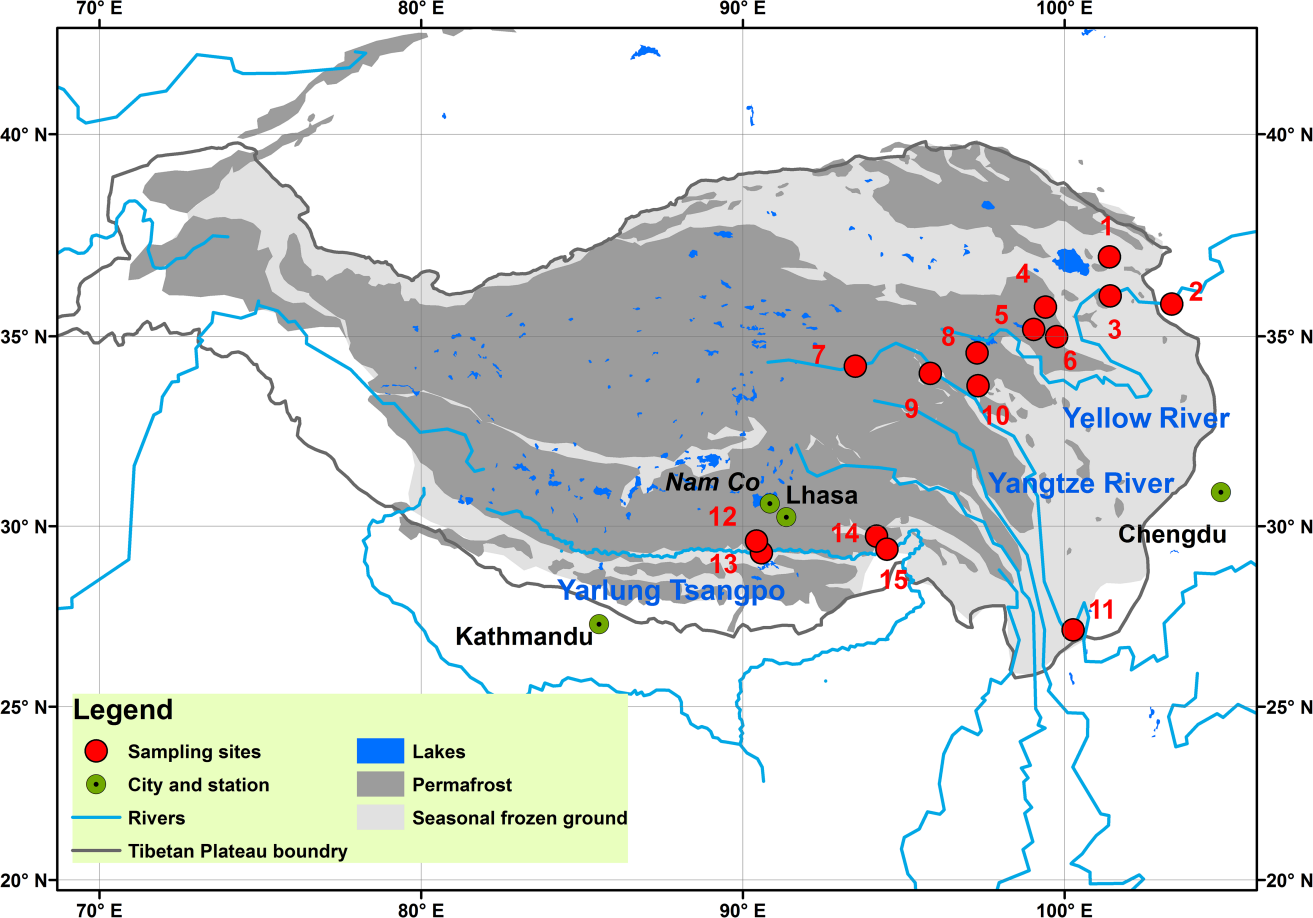
Permafrost distributions, locations of sampling sites and stations with precipitation data for the headwater rivers of the Tibetan Plateau. Data of permafrost distributions were from [20]. The map was plotted by ArcGIS 10.2.1 software (ESRI^®^). Detailed information concerning the sampling sites is shown in Table 1.

There are three isotopes of carbon on Earth: 99% of the carbon is carbon-12 (^12^C), 1% is carbon-13 (^13^C), while carbon-14 (^14^C) occurs in trace amounts, i.e., making up about 1 or 1.5 atoms per 1012 atoms of the carbon in the atmosphere (0.0000000001%) [15, 16]. ^12^C and ^13^C are stable carbon isotope and ^13^C has been widely used in source identifications in global carbon study [17]. With a half-life of 5568±30 years [18], radiocarbon ^14^C were widely employed to be a tool for the age dating in geology and archaeology [19]. More ^14^C depletion means older age of DOC [17]. There has been a surging usage of ^14^C in the last decades with the increasing accessibility of accelerator mass spectrometry (AMS) facilities and higher measurement accuracy [19]. Therefore, along with Δ^14^C, we can provide additional resolution where δ^13^C signatures were overlaid by different sources [1]. Here we built an extensive new set of Δ^14^C data on global riverine DOC pools from three large river basins (the Yellow, Yangtze and Yarlung Tsangpo Rivers, Fig 1) on the Tibetan Plateau, a continent from which no ^14^C data are currently reported. In addition, the potential source of DOC in the rivers of the Tibetan Plateau and its relationship with thawing permafrost were also discussed. The combined data set of DOC concentrations and carbon isotopic compositions (Δ^14^C and δ^13^C) outlined in rivers of the Tibetan Plateau in this study can be used to explore the global ^14^C signatures of riverine carbon pools in different environmental patterns and to draw attention to some of the factors driving their variability.

## Materials and methods

River water from the Yellow, Yangtze and Yarlung Tsangpo Rivers was sampled during a high flow period in 2014 (Fig 1 and Table 1). At each site, one liter of water was collected with acid-leached polycarbonate bottles and the samples were filtered through pre-combusted (450°C for 6 h) 47 mm diameter (GF/F 0.7 μm) glass fiber filters in the field [21]. The filtered samples were frozen at -20°C after collection until being analyzed.

**Table 1:** Sampling information of the studied rivers.

	Latitude (N)	Longitude (E)	Elevation (m)	Permafrost/total area (%)	River Type [25]	Sample site	Sampling date
Yellow River	36°54.302′	100°59. 926′	3,006	24.06	T	1	2014/08/12
	36°08.238′	103°36. 577′	1,525	33.68	M	2	2014/08/19
	36°02.502′	101°24. 316′	2,196	28.49	M	3	2014/08/13
	35°43.306′	99°32. 847′	3,796	47.27	S	4	2014/08/14
	35°00.774′	98°04. 050′	4,241	63.70	S	5	2014/08/15
	35°03.939′	98°42. 220′	4,475	100	S	6	2014/08/18
Yangtze River	34°13.368′	92°26.308′	4,540	100	M	7	2014/09/12
	34°05.963′	97°37. 847′	4,701	100	S	8	2014/08/16
	32°58.924′	97°14. 054′	3,520	70	T	9	2014/08/17
	32°59.646′	97°14. 963′	3,521	94.94	M	10	2014/08/16
	26°53.896′	100°01.430′	1,824	n.d.	M	11	2014/09/03
Yarlung Tsangpo	29°21.952′	90°51.915′	3,595	0	T	12	2014/08/23
	29°16.649′	90°48.615	3,585	9.31	M	13	2014/08/23
	29°26.862′	94°27.022′	2,932	2.22	T	14	2014/08/25
	29°21.325′	94°24.046′	2,927	12.20	M	15	2014/08/28

“M” “T” and “S” stand for sampling in “main stream”, “tributary” and “small stream”, respectively.

DOC concentration was determined using a Shimadzu TOC-5000 total organic carbon analyzer (Shimadzu Corp, Kyoto, Japan) in the Key Laboratory of the Tibetan Environment Changes and Land Surface Processes, Institute of Tibetan Plateau Research [22].

The pretreatment for determination of carbon isotope compositions (Δ^14^C and δ^13^C) was conducted at Yale University following published methods [17, 21, 23] with minor modifications. In brief, water samples were acidified to pH 2.5 with 40% (V/V) phosphoric acid and sparged with ultra-high purity Helium gas for 10 min to remove inorganic carbon, then the samples were irradiated with a medium pressure mercury arc UV lamp of 2400 W (Canrad-Hanovia, Newark, NJ) for 5 h. Compared with DOC concentrations measured with high-temperature oxidation method, which was performed on a Shimadzu TOC analyzer and UV oxidation method, we found the conversion efficiency of DOC after the UV exposure [21] can be as high as 102±3% in this study. The CO_2_ generated from DOC oxidation was purified and collected on a vacuum extraction line. Concentrations of DOC were determined using a calibrated Baratron absolute pressure gauge (MKS Industries) to measure CO_2_ pressure. Following quantification, the sample was split approximately 10:1 to two break-seal tubes, with the larger portion being used for ^14^C analysis and the smaller portion being used for ^13^C analysis. Recoveries and blanks were assessed periodically by oxidizing a standard of dissolved organic standard (oxalic acid) using the same procedure as for samples. The ^14^C analyses of trapped CO_2_ were performed by accelerator mass spectrometry (AMS) at the National Ocean Sciences AMS at Woods Hole Oceanographic Institution. Δ^14^C values of the standard were within analytical precision of the accelerator mass spectrometer. A sample from “Modern” is defined as 95% of the radiocarbon concentration (in AD 1950) of NBS oxalic acid normalized to δ^13^C_VPDB_ = -19 ‰ [24].

Permafrost, watershed area and reliefs of the rivers were extracted from ArcGIS 10.2.1 software (ESRI^®^). Watershed area of rivers was downloaded from International Scientific & Technical Data Mirror Site (http://www.gscloud.cn), Computer Network Information Center, Chinese Academy of Sciences. Permafrost area data was developed by State Key Laboratory of frozen soil engineering, Chinese Academy of Sciences [20].

## Results and discussion

### Export of DOC in rivers of the Tibetan Plateau

The average DOC concentrations in the Yellow, Yangtze and Yarlung Tsangpo Rivers on the Tibetan Plateau were 2.09±0.41 mg-C L^-1^, 1.88±0.81 mg-C L^-1^ and 1.16±0.31 mg-C L^-1^, respectively, much lower than average values for tropical (6.69±0.36 mg-C L^-1^), temperate (4.40±0.16 mg-C L^-1^) and Arctic rivers (6.12±0.23 mg-C L^-1^) [21, 25]. Soil organic carbon (SOC) density in the river catchments can positively affect the riverine DOC [26]. That means, rivers running on an area with higher density of SOC usually have higher DOC concentrations. However, it was estimated that SOC in Tibetan soils was only 6.5 kg m^-2^, much lower than that in the Arctic Alaska and most other tropical and temperate regions in the world (Table A in S1 File) [27–29]. The low level of DOC concentrations on the rivers of the Tibetan Plateau might be also due to the DOC in Tibetan rivers is labile and more readily attenuated. Here we measured the ratio of the DOC and dissolved organic nitrogen (DOC/DON, C/N) in the waters of the plateau, which is usually used as an index for the bioavailability of dissolved organic matter [30]. The average C/N in the selected rivers on the Tibetan Plateau was ~8.1 (Table 2), much lower than the global average of 22.1 [31]. Low C/N ratio usually means that dissolved organic matter is more easily decomposed into CO_2_ [32]. In addition, it was reported that most riverine DON was derived from leaching and erosion of the land and contained low molecular weight of organic nitrogen with high bioavailability [33], suggesting that a large proportion of carbon in the rivers of the Tibetan Plateau was available for biological metabolism [30]. Therefore, when compared with the large rivers in Arctic, although the mean annual water flux in the drainage area of the three rivers on the Tibetan Plateau was comparable or higher than the average for large Arctic rivers [21], the mean annual DOC yield from the basins of these three rivers on the plateau (0.41±0.19 g-C m^-2^ yr^-1^) was much lower than the DOC yields of large Arctic rivers to the Arctic Ocean (1.6 g-C m^-2^ yr^-1^) [21].

**Table 2:** Discharge (km^3^ yr^-1^), dissolved organic carbon (DOC) concentration (mg-C L^-1^), C/N (unitless) and DOC loads (GgC yr^-1^) of three rivers draining the Tibetan Plateau.

Region	Rivers	Discharge of water	DOC	C/N	Flux of DOC
Third Pole	Yellow river	31.3	2.09	5.1	65.4
	Yangtze river	44.5	1.88	8.6	83.7
	Yarlung Tsangpo [34]	139.5	1.16	10.6	161.9

Note: Discharge data for the Yellow River and Yangtze River are from Lanzhou (sample 2 at Fig 1) and Shigu hydrologic station (sample 11 at Fig 1) and were adopted from the Bureau of Hydrology, Ministry of Water Resources, China.

The average DOC concentrations of these three rivers increased with increasing rainfall and temperature from northwest to southeast [35]. The rainfall and temperature largely influence the vegetation cover and soil organic carbon on the land surface of the Tibetan Plateau [36]. There spreads high proportion of grasslands and even forests in the southeast plateau, while in the central and northwest of the Tibetan Plateau, there were dominated by alpine vegetation (Figure A in S1 File). The different SOC storage in the different vegetation soils [27, 37, 38] lead to the higher DOC concentrations in the southeastern plateau. Based on discharge and DOC concentrations measured at stations at the fringe of the Tibetan Plateau, the estimated DOC load exported out from the plateau by these three rivers totaled 0.31 Tg-C (1 Tg = 10^12^ g) (Table 2).

### DOC carbon isotopes compositions in rivers of the Tibetan Plateau

**Fig 2:**
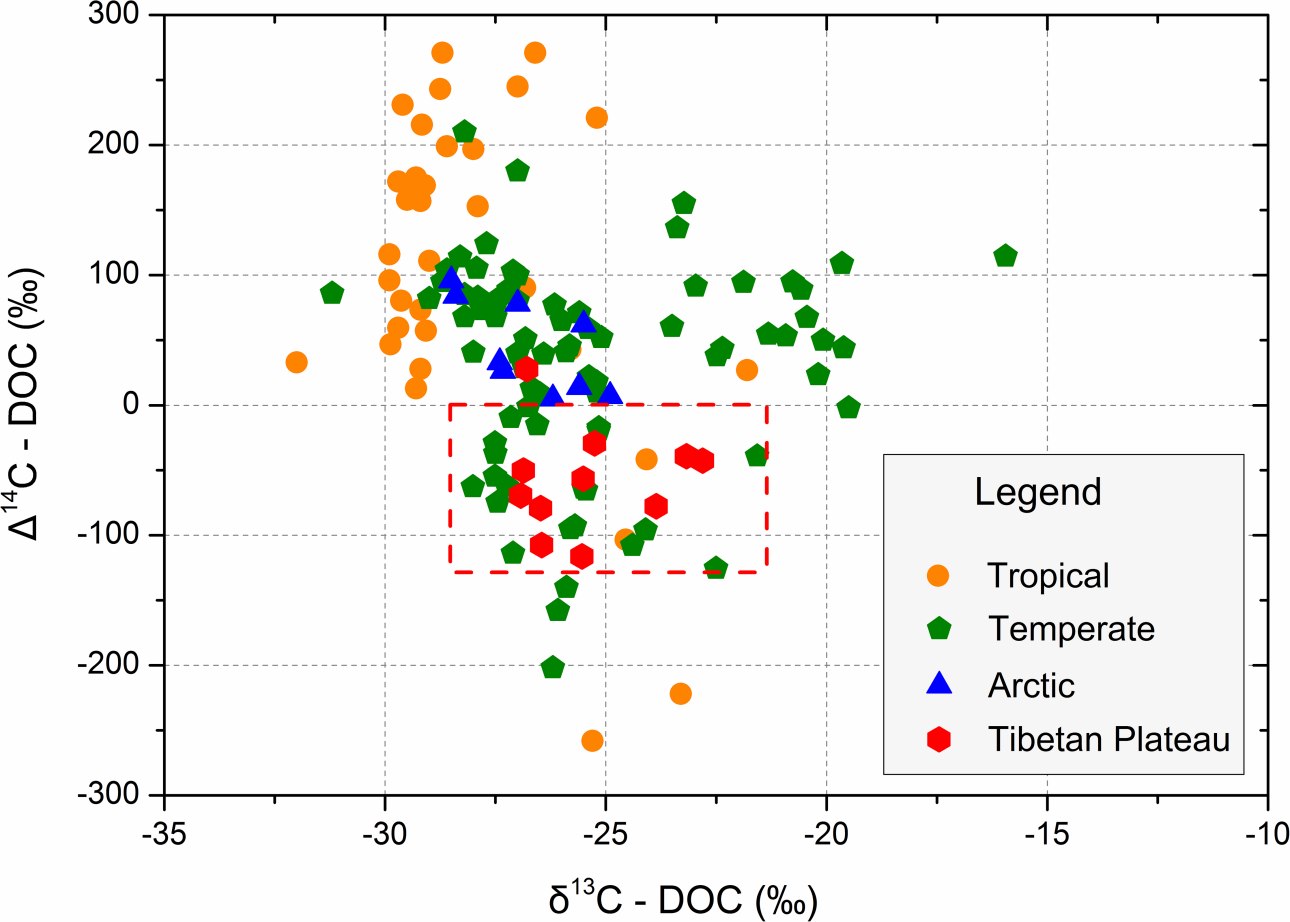
Distributions of Δ^14^C and δ^13^C isotopes of dissolved organic carbon for rivers of the Tibetan Plateau and other regions. A full reference list for the carbon isotopes data is provided in Data Set 1.

The average measured δ^13^C values in the headwaters of the Yellow River, the Yangtze River and the Yarlung Tsangpo were -25.8±1.5 ‰, -25.9±0.7 ‰ and -25.1±1.8 ‰, respectively. These values are similar (slightly depleted) to δ^13^C values measured in Tibetan surface soils [39] and lie within the range of δ^13^C-DOC values for rivers in other regions (Fig 2), reflecting typical values for terrestrial organic matter [23]. The mean ^14^C-age of the DOC in rivers of the Tibetan Plateau was 511±294 (yrs BP). The youngest DOC age was identified in a tributary of the Yarlung Tsangpo (the Niyang River, Site 14, Table 3), a densely-forested river in the southern Tibetan Plateau that experiences relatively warm and humid weather. DOC from the Yarlung Tsangpo (310±347 yrs BP) was younger than from the Yellow (539±220 yrs BP) and Yangtze Rivers (669±289 yrs BP). The latter two river catchments have greater permafrost coverage (Fig 1; Table 1).

**Table 3:** Concentrations, stable carbon and radiocarbon isotope values for dissolved organic carbon in Tibetan Plateau rivers.

River	Sample	DOC (mg-C L^-1^)	Radiocarbon age (yr BP ± stdev)	Δ^14^C (‰ ± stdev)	δ^13^C (‰)
Yellow River	1	1.96	350 ± 20	-50.2 ± 3	-26.9
	2	2.27	420 ± 15	-58.3 ± 2	n.d.
	3	2.09	955 ± 20	-119 ± 2	n.d.
	4	2.60	405 ± 15	-56.6 ± 2	-25.5
	5	1.37	590 ± 15	-77.8 ± 2	-23.9
	6	2.22	515 ± 20	-69.6 ± 3	-26.9
Yangtze River	7	0.99	855 ± 25	-108 ± 3	-26.5
	8	2.28	600 ± 15	-79.2 ± 2	-26.5
	9	3.04	930 ± 25	-116 ± 3	-25.5
	10	1.33	290 ± 20	-42.7 ± 3	n.d.
	11	1.79	n.d.	n.d.	-25.1
Yarlung Tsangpo	12	1.29	260 ± 25	-39.2 ± 4	-23.2
	13	1.01	175 ± 20	-29.3 ± 3	-25.3
	14	1.53	modern	27.8	-26.8
	15	0.82	805 ± 30	-102.0 ± 4	n.d.

n.d: no data

### Potential sources of old carbon in rivers of the Tibetan Plateau

Compared to DOC from rivers in other regions of the world, DOC from the Tibetan rivers was rather depleted in ^14^C and represented an old carbon pool to the global carbon budget [40] (Fig 2). For instance, DOC ages in these Tibetan rivers were older than those in Arctic and tropical rivers (Fig 2). Furthermore, when considering high flow periods that dominate export to the coast, Arctic systems are generally ^14^C enriched compared to the atmosphere [21], while Tibetan rivers are 511 yrs BP. DOC ages in large tropical rivers are generally young due to inputs of near-modern soil and biomass carbon from the mostly pristine forests and wetlands that dominate their catchments [41]. In temperate rivers, DOC becomes older with increased human population density and higher ratios of human-dominated landscapes within a basin[25, 42]. The population density and farmland ratio of the Tibetan Plateau (5.6 people km^-2^ and 0.5%, respectively) is very low [43]. Furthermore, the small amount of residential and farm land that does exist is distributed along the fringes of the plateau, with even lower population densities and human activities at the higher elevations of the inhospitable central Tibetan Plateau [44]. Therefore, human activities on the Tibetan Plateau should have little influence on the supply of old DOC to rivers.

**Fig 3:**
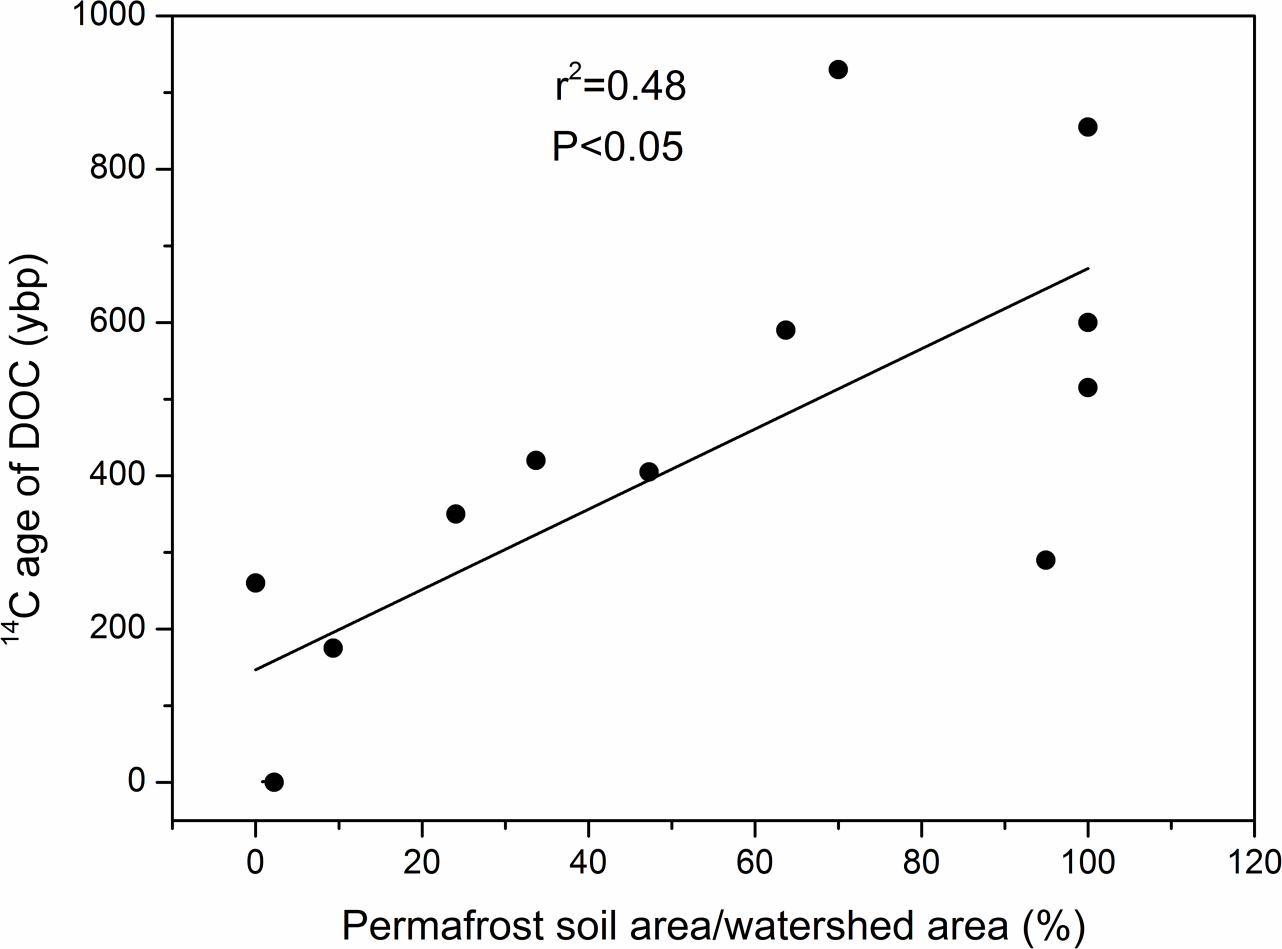
Significant relationship between permafrost soil area as a percentage of watershed area (%) and ^14^C age of dissolved organic carbon (DOC) in years before present. Note: samples 3 and 15 were not included in the regression because the old DOC ages of these two samples were attributed to factors other than permafrost carbon contributions. Sample 3 was collected from the lower reaches of the large Longyangxia reservoir on the main stem of the Yellow river. The long residence time of water in the reservoir [23] or carbon age reservoir effects [50] may therefore have contributed to the old ^14^C age of DOC in this sample. Sample 15 was collected from Yarlung Tsangpo Grand Canyon, the region with the maximum topographic slope on the Tibetan Plateau [51].

The depleted ^14^C implying a greater contribution from old carbon sources. Different with rivers flowing in tropical and temperate regions, most rivers running on the Tibetan Plateau flow over an extensive permafrost soil coverage and with no intensive human activities (Fig 1). The percentage coverage of permafrost soil within river catchments on the Tibetan Plateau was positively correlated with DOC ^14^C-age (r^2^ = 0.48, P<0.05) (Fig 3), suggesting that the old DOC in rivers of the plateau may be due to the prevalence of ancient permafrost soil carbon underlying much of the river catchments in the region. The older ages may be facilitated by a climate warming induced deep active layer [14] interacting with summer monsoon rains. Changes in stream DOC export due to climate warming have been observed in Arctic peatland watersheds [45]. Discharge patterns drive DOC export in many rivers. In the Arctic, the highest discharges and DOC fluxes occur during spring floods, when the surface soils are frozen [21]. In contrast, the Tibetan Plateau experiences a strong monsoonal rain pattern. The monsoon rains fall when the soil active layer depth is near its maximum [46], potentially mobilizing older peat carbon to streams and rivers. Interestingly, the DOC ages in Arctic rivers presented a negative correlation with the permafrost coverage [47], which was opposite to that of the rivers on the plateau. We address this to two reasons, first, with temperature increasing, the ALT on the Tibetan Plateau has been increased up to 2.41 m [48], much larger than that in Arctic river basins (~1.74 m) [49]; Second, different with that in Arctic basins, the monsoonal rainfalls on the Tibetan Plateau occur during times when the active soil layer is the deepest [21]. Therefore, with the larger rainfalls and deeper ALT at the same time, the rivers flows on the Tibetan Plateau will carry more old carbon from areas with larger permafrost coverage. Furthermore, the deepening of the permafrost active layer on the Tibetan Plateau could lead to new hydrologic flow paths and interactions of water with ancient stored carbon.

The geomorphic and hydrologic characteristics of river basins also have an important effect on the absolute ages of riverine DOC [23, 52]. The reliefs of the three studied river basins are 0.21%, 0.28% and 0.16%, respectively. These values are much higher than those of most other rivers in the world [53]. It is likely that the high relief of the studied river basins could lead to high velocity of water flow, and result in intense mechanical weathering, releasing deeper and older soil and carbon into the rivers. Therefore, in addition to high permafrost carbon coverage, the high relief of the studied river basins may enhance the export of ancient DOC.

It is currently difficult to determine the exact source of old DOC to the Tibetan river waters. However, it is clear that rivers export old carbon from permafrost regions of the Tibetan Plateau during the monsoon. This is distinct from permafrost regions in the Arctic, where ancient permafrost-derived DOC does not have a measurable impact upon the bulk radiocarbon age of the DOC in larger rivers [21], likely due to a larger amount of young bomb carbon enriched DOC “hiding” the old carbon in the Arctic and potentially a smaller old component during high discharge periods due to a shallower active layer in the Arctic.

The results of this study represent the first isotope-DOC dataset for large rivers on the Tibetan Plateau. The thawing of permafrost and soil erosion caused by the high relief on the plateau may contribute to the old ages of the riverine DOC. If old DOC in rivers of the plateau derives mainly from the mobilization of permafrost carbon during the monsoon, then the export of ancient DOC may increase as the responds to climate change-induced permafrost thaw and shifts in rainfall. Once the previously frozen permafrost DOC is released into surface waters, it will likely be rapidly degraded by sunlight and microbial respiration, resulting in CO_2_ emission back to the atmosphere, producing a positive feedback on climate warming [3, 54, 55]. The influx of solar radiation, especially at UV wavelengths, is very strong on the Tibetan Plateau compared to other regions, due to the thin air, high altitudes and low latitudes [56]. Hence, the reaction of previously frozen DOC to this uniquely intense solar irradiance needs to be comprehensively studied to assess the fate of the DOC in rivers on the Tibetan Plateau.

## Supporting information

S1 File Supporting material for “Aged dissolved organic carbon exported from rivers of the Tibetan Plateau”. (PDF)

S2 File Dataset of Fig 2. (XLSX)
