# Correction: Aged dissolved organic carbon exported from rivers of the Tibetan Plateau

**DOI:** 10.1371/journal.pone.0181295

**Published:** 2017-07-07

**Authors:** Bin Qu, Mika Sillanpää, Chaoliu Li, Shichang Kang, Aron Stubbins, Fangping Yan, Kelly Sue Aho, Feng Zhou, Peter A. Raymond

There are errors in the Funding section. The correct funding information is as follows: This work was funded by the National Nature Science Foundation of China (41675130, 41630754), State Key Laboratory of Cryospheric Science (SKLCS-ZZ-2017), the Academy of Finland (decision number 268170).

Additional affiliations for the first and third authors are missing. Bin Qu is affiliated with both **1** Laboratory of Green Chemistry, Lappeenranta University of Technology, Mikkeli, Finland and **3** Key Laboratory of Tibetan Environment Changes and Land Surface Processes, Institute of Tibetan Plateau Research, Chinese Academy of Sciences, Beijing, China. Chaoliu Li is affiliated with both **3** Key Laboratory of Tibetan Environment Changes and Land Surface Processes, Institute of Tibetan Plateau Research, Chinese Academy of Sciences, Beijing, China and **5** CAS Center for Excellence in Tibetan Plateau Earth Sciences, Chinese Academy of Sciences, Beijing, China.
