# Supplementary material for: Aged dissolved organic carbon exported from rivers of the Tibetan Plateau
Source: PLoS One. 2017 May 26;12(5):e0178166. doi: 10.1371/journal.pone.0178166 (PMC5446113; doi:10.1371/journal.pone.0178166)
Supplement: S1 File — (PDF) [file pone.0178166.s001.pdf]

Supporting Material for

Aged dissolved organic carbon exported from rivers of the Tibetan Plateau

Bin Qu[1], Mika Sillanpää[1,7], Chaoliu Li[2]*, Shichang Kang[3,4], Aron Stubbins[5], Fangping Yan[1], Kelly Sue Aho[6], Feng Zhou[8] and Peter A. Raymond[6]

[1] Laboratory of Green Chemistry, Lappeenranta University of Technology, Mikkeli, Finland

[2] Key Laboratory of Tibetan Environment Changes and Land Surface Processes, Institute of Tibetan Plateau Research, Chinese Academy of Sciences, Beijing, China

[3] State Key Laboratory of Cryospheric Sciences, Norwest Institute of Eco-Environment and Resources, Chinese Academy of Sciences, Lanzhou, Gansu, China

[4] CAS Center for Excellence in Tibetan Plateau Earth Sciences, Chinese Academy of Sciences, Beijing, China

[5] Skidaway Institute of Oceanography, Department of Marine Science, University of Georgia, Savannah, USA

[6] Yale School of Forestry and Environmental Studies, Yale University, New Haven, Connecticut, USA

[7] Department of Civil and Environmental Engineering, Florida International University, Miami, Florida, USA

[8] Sino-France Institute of Earth Systems Science, Laboratory for Earth Surface Processes, College of Urban and Environmental Sciences, Peking University, Beijing, China

* Corresponding author

E-mail: lichaoliu@itpcas.ac.cn (CL)

Contents

1. Supporting text

1.1 Climate and Environment of the Tibetan Plateau

1.2 Seasonal Variations of DOC Concentration and Flux

2. Supporting figures

Figure A. Spatial distributions of the vegetation cover on the Tibetan Plateau.

Figure B. Spatial distributions of annual precipitation (mm) during 2011 to 2013 of the Tibetan Plateau.

Figure C. Relationship between monthly precipitation and discharge of inland Tibetan Plateau catchments and rivers.

Figure D. Comparison between dissolved organic carbon (DOC) concentrations during the monsoon and non-monsoon period of the selected rivers.

3. Supporting tables

Table A. Estimated of soil organic carbon (SOC) in different areas of the world.

4. References for supporting online material

1. Supporting text

1.1 Climate and Environment of the Tibetan Plateau

Precipitation patterns across the Tibetan Plateau are detailed in Figure B. The Tibetan Plateau is influenced by both westerlies and the Asian monsoon. Annual climate is characterized by a cool, dry winter, and a wet, humid summer [1]. About 60–90% of the annual precipitation to the Tibetan Plateau occurs from June to September [2]. The moisture is transported from the Indian and Pacific Ocean by Indian and East Asia monsoons, respectively [3]. Peak discharge in the Tibetan rivers occurs during the monsoon and correlates significantly with variations in precipitation (Fig. S3). Because the precipitation is transported mainly from south and east of the plateau, the mean annual precipitation also decreases from south-east to north-west [4].

1.2 Seasonal Variations of DOC Concentration and Flux

Annual discharge from the rivers on the Tibetan Plateau is dominated by the monsoon, with 43% of discharge occurring during June to August of the year, and DOC concentration is similar between the monsoon and non-monsoon seasons [5] (Fig. S4). Accordingly, DOC export from the three rivers was estimated based on DOC concentration during the monsoon period annual water discharge. The annual water discharged out of the Tibetan Plateau by the Yellow River, the Yangtze River and the Yarlung Tsango (Fig. 1) was around 31.3, 44.5 and 139.5 km^3 yr^{-1}, respectively. Therefore, annual DOC transported out of the Tibetan Plateau by these three rivers was around 66.2 GgC yr^{-1}, 84.9 GgC yr^{-1} and 161.9 GgC yr^{-1} ($Gg=10^9g$) (Table S3).

2. Supporting figures

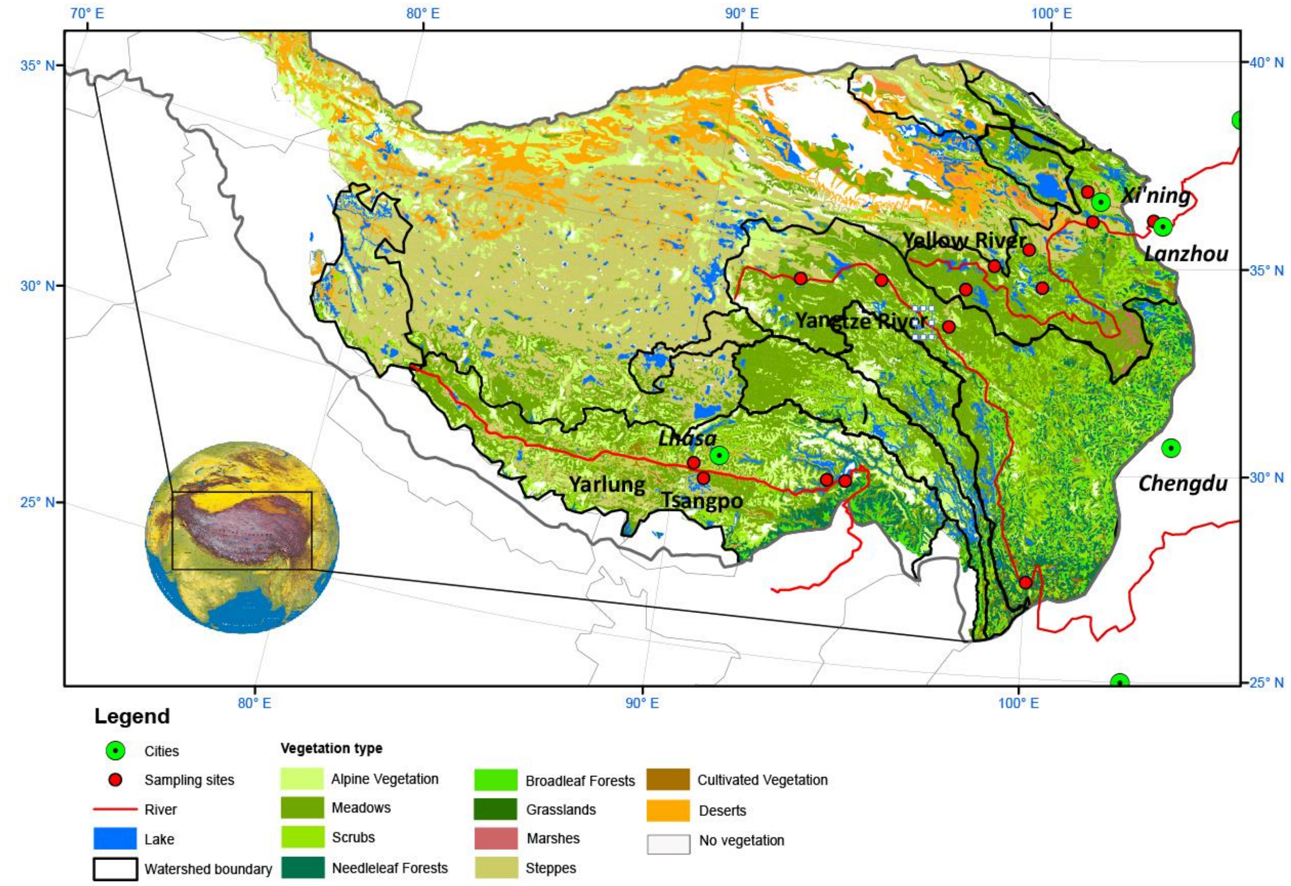

Figure A. Spatial distributions of the vegetation cover on the Tibetan Plateau. Map was adopted from Vegetation atlas of China [6] and processed with ArcGIS 10.0 (ESRI®)

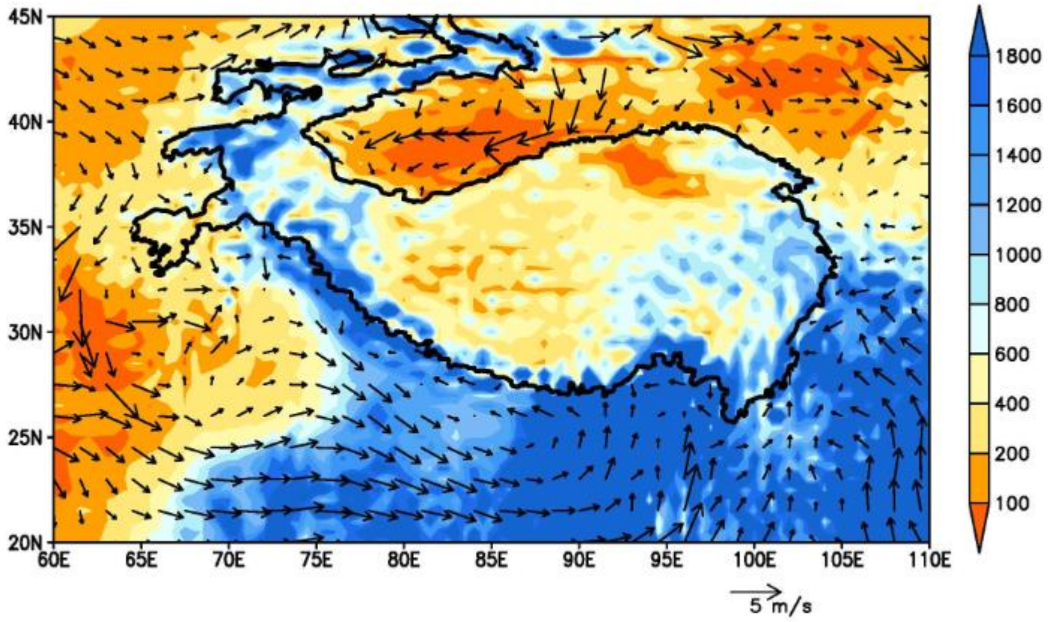

Figure B. Spatial distributions of annual precipitation (mm) during 2011 to 2013 of the Tibetan Plateau. Note: data of precipitation and wind field data were adopted from Global Precipitation Climatology Project (GPCP) and National Centers for Environmental Prediction (NCEP), respectively.

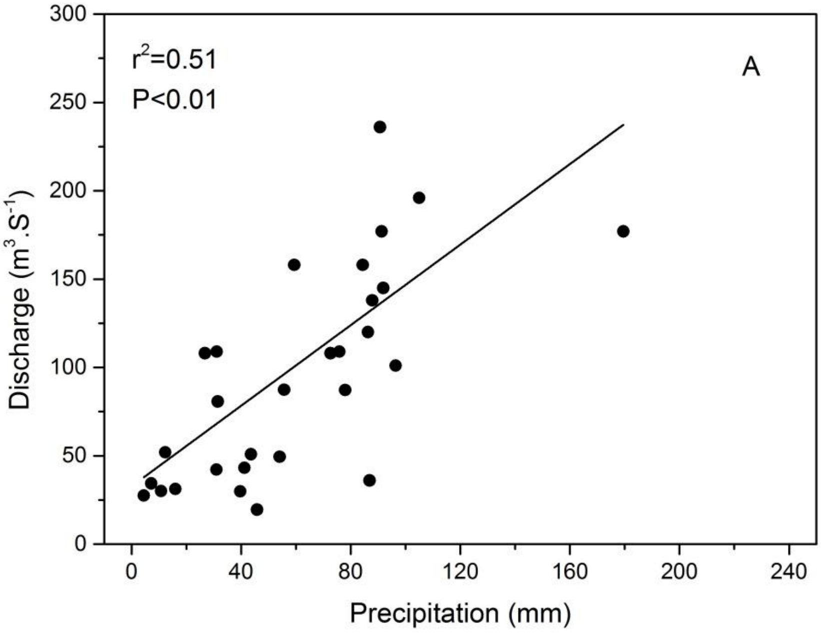

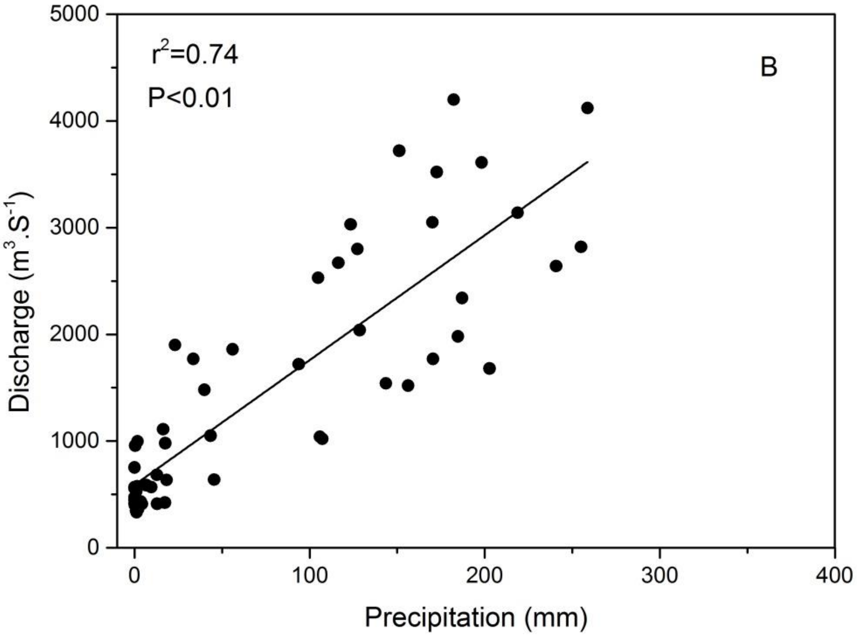

Figure C. Relationship between monthly precipitation and discharge of inland Tibetan Plateau catchments and rivers (A: precipitation and discharge at Tuotuohe) and fringe Tibetan Plateau catchments and rivers (B: precipitation at Lijiang and discharge at Shigu, respectively) during 2008 and 2012.

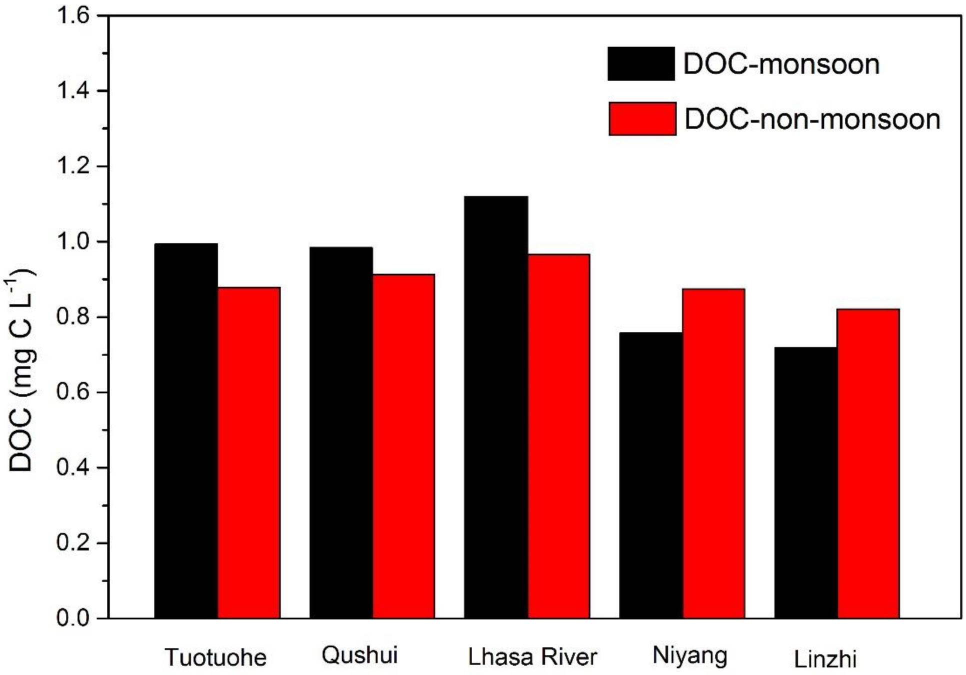

Figure D. Comparison between dissolved organic carbon (DOC) concentrations during the monsoon and non-monsoon period of the selected rivers.

2. Supporting tables

Table A. Estimated of soil organic carbon (SOC) in different areas of the world.

Soils, locations	Mean SOC density	Pedons	Coefficient of variatiion
	kg m^{-2}	no.	%
**Alaska** [7]			
Cryofolists	14.2	4	30
Deep Entisols	32.4	7	34
Deep Spodosols	29.8	95	35
Shallow Entisols	16.9	7	28
Shallow Spodosols	17.4	26	42
**Global** [8]			
Tropical Oxisols	9.7	71	42
Tropical Ultisols	8.3	53	70
Temperate Alfisols	5.5	354	62
Temperate Mollisols	9.1	522	46
Aridisols	4.2	98	60
**Tibetan Plateau** [9]			
Alpine meadow	9.1	57	69
Alpine steppe	4.4	78	79

3. References for supporting online material

1. Yang K, Wu H, Qin J, Lin C, Tang W, Chen Y. Recent climate changes over the Tibetan Plateau and their impacts on energy and water cycle: A review. Global and Planetary Change. 2014;112:79-91.

2. Wei Z, Huang R, Dong W. Interannual and interdecadal variations of air temperature and precipitation over the Tibetan Plateau. Chinese Journal of Atmosphere Sciences-Chinese Edition. 2003;27(2):157-70.

3. Xu X, Zhang R, Shi X, Zhang S, Bian L, Cheng X, et al. A new integrated observational system over the Tibetan Plateau. Bulletin of the American Meteorological Society. 2008;89(10):1492-6.

4. You Q, Min J, Zhang W, Pepin N, Kang S. Comparison of multiple datasets with gridded precipitation observations over the Tibetan Plateau. Climate Dynamics. 2014:1-16.

5. Cao J, Qin D, Kang E, Li Y. River discharge changes in the Qinghai-Tibet Plateau. Chinese Science Bulletin. 2006;51(5):594-600.

6. Hou X. Vegetation atlas of China. Chinese Academy of Science, the editorial board of vegetation map of China. 2001.

7. Alexander E, Kissinger E, Huecker R, Cullen P, editors. Soils of southeast Alaska as sinks for organic carbon fixed from atmospheric carbon-dioxide. Proc of Watershed'89: A conference on the Stewardship of Soil, Air, and Water Resources; 1989.

8. Kimble J, Eswaran H, Cook T, editors. Organic carbon on a volume basis in tropical and temperate soils. Transactions 14th International Congress of Soil Science, Kyoto, Japan, August 1990, Volume V; 1990.

9. Yang Y, Fang J, Tang Y, Ji C, Zheng C, He J, et al. Storage, patterns and controls of soil organic carbon in the Tibetan grasslands. Global Change Biology. 2008;14(7):1592-9.
